# Diphtheria

**Published:** 1899-11-04

**Authors:** P. Watson Williams

**Affiliations:** Physician to Clifton College; and Physician in Charge of the Department for Diseases of the Throat, Bristol Royal Infirmary.


### Hospital Clinics and Medical Progress.

### DIPHTHERIA.

By P. Watson Williams, M.D.Lond., Physician to Clifton College; and Physician in Charge of the Department for Diseases of the Throat, Bristol Royal Infirmary.

(*Continued from page* 60.)

**Serum Treatment.**—It may be fairly said that diphtheria has been largely robbed of its terrors since the introduction of the antitoxic serum, and there are very few practitioners who have any doubt of its enormous value in controlling the disease, and, given early enough, completely changing for the better the clinical course of the great majority of cases. It is essential that the antitoxin be administered as early as possible, its efficacy in controlling the disease and reducing the mortality decreasing with each day that elapses between the onset of the disease and the commencement of the injections. This is the almost universal experience, a fact which demonstrates very forcibly the value of the antitoxin and the limitations of its usefulness.

The dose of antitoxin must not be too small. An average amount for an adult is 8,000 units in the first twelve hours, and after that from 3,000 to 2,000 units daily until the membrane disappears. If the attack appears to be particularly virulent, or if the case be not brought under treatment before the third day it is desirable to increase the initial dosage to 12,000 units, followed by 2,000 to 8,000 units every 12 hours for 48 hours; whereas, in mild cases seen quite early in the attack, smaller amounts may prove equally effective. It is better to err on the side of an overdose, inasmuch as the antitoxin itself is practically harmless, though certain complications may arise from throwing considerable amounts of animal serum into the circulation, wherefore a serum of high potency is better than one containing relatively few antitoxic units per cubic centigramme.

Behring,[38] experimenting on the absorption and excretion of antitoxin, found that the antitoxin is gradually excreted, and that an increased dose causes a disproportionate increase in the rate of excretion. It is, therefore, probably better to divide the larger initial doses; and, in attempting to prolong the period of immunity, it is better to repeat the dose rather than to increase its strength indefinitely.

Fisch[39] has demonstrated by experiments on animals, on himself, and on friends that artificial immunity may be induced by the administration by the mouth of antitoxic serum, or of milk from a cow or a goat artificially immunised to a high degree. But it takes from 24 to 36 hours for the complete absorption and diffusion of the antitoxin through the body, for even in 11 hours it did not render the blood serum antitoxic to an appreciable degree.

The records of the University College cases reported by Martin and Hunt[40] are most instructive. All the cases were admitted on clinical grounds as diphtheria, thus disposing of any fallacious comparison on the score of the mild cases which were formerly overlooked clinically being included solely on the bacteriological test. In all there were admitted in 1896 and 1897 a total of 185 cases, but of these seven must be excluded (viz., five which recovered without antitoxin because they were not considered to be true diphtheria, and two fatal cases in which antitoxin was only given by the mouth), leaving 178 cases treated with antitoxin. The effect on the mortality is best shown by comparing the mortality of the three antitoxin years with the preceding non-antitoxin years. The percentage mortality in the four non-antitoxin years was, laryngeal cases, 70, 68, 78, 47; pharyngeal cases, 18·7, 14·3, 10·9, 30·0; total percentages, 43·5, 33·3, 37, 39. The same figures in the three antitoxin years successively were, laryngeal cases, 33·3, 40·0, 23·5; pharyngeal, 24·4, 10·0, 13·0; total percentages, 28·0, 17·7, 17·0. Again, they compare the mortality in the tracheotomy and intubation cases.

The effect of antitoxin upon the incidence of those complications which commonly occur in diphtheria is fairly shown by the following table compiled by Michael, drawn from the reports of the Metropolitan Asylums Board:—

Complications Occurring in all Cases.

| | 1894. No Antitoxin. | | 1895. Antitoxin. | | 1896. Antitoxin. | |
|---|---|---|---|---|---|---|
| | No. of Cases. | Per cent. on total. | No. of Cases. | Per cent. on total. | No. of Cases. | Per cent. on total. |
| Albuminuria ... | 603 | 24·1 | 1,081 | 40·9 | 1,663 | 60·1 |
| Nephritis ... ... | 37 | 1·2 | 45 | 2·0 | 16 | ·5 |
| Paralysis ... ... | 403 | 13·2 | 507 | 23·2 | 713 | 25·7 |
| Lobar Pneumonia | 11 | ·3 | 18 | ·8 | 19 | ·6 |
| Lobular ,, | 50 | 1·6 | 80 | 3·6 | 86 | 3·1 |
| Relapse ... ... | 28 | ·9 | 31 | 1·4 | 52 | 1·8 |

It will be seen that there is a decided increase in the cases of albuminuria and paralysis, and these facts demand further consideration.

**The Action of Antitoxic Serum on the Blood Corpuscles** has been investigated by J. S. Billings, jun.,[41] Kanthack and Lloydd,[42] and others. They found that the antitoxin treatment of diphtheria has no deleterious effects upon the blood corpuscles, but that, on the contrary, it seems to prevent degenerative changes that otherwise occur in the course of this disease, Billings more especially noting that the diminution of red corpuscles is much less marked than in those cases treated without it.

**The Connection between Antitoxin Serum and Albuminuria.**—Personally I have never been able to trace any connection between the serum injections and nephritis, but certainly it does appear to cause transient albuminuria. This question has been investigated both clinically and experimentally by Siegert.[43] He finds that after the injections there occurs generally a slight transitory albuminuria and albumosuria; this was found, not only in patients already suffering from diphtheria, but also in healthy children in whom the antitoxin was injected as a prophylactic measure. In animals the injection produced similar change in the urine and also a diminution in its quantity and specific gravity. In some cases acute parenchymatous and hæmorrhagic nephritis occurred in patients treated with the serum, but there was no evidence that this occurred unless some change had taken place in the serum used. Occasionally anuria occurred after the injection, and the same phenomenon was observed in animals. If albuminuria be already present in a case of diphtheria the injection of antitoxin generally causes the albuminuria to disappear without evil consequences. That the alterations in the urine are generally due to mere functional disturbance in the kidney seems to be shown by the fact that even with 10 c.cm. of Behring's serum no organic lesion of the kidney could be produced in rabbits.

Goodall[44] records three cases of post-scarlatinal nephritis at the Eastern Fever Hospitals. In cases 1 and 2 the patients were actually suffering from nephritis when the serum was injected, and the renal inflammation was in no way aggravated; quite the contrary, in fact, in case 2. In case 3 the patient had apparently recovered from the nephritis, but there was a recurrence of the nephritis subsequent to the injection of the antitoxin.

[38] Fortsh. der Med., Jan., 1897; Brit. Med. Jour., Feb. 6, 1897. [39] New York Med. Rec., April, 1898. [40] Brit. Med. Jour., May 29, 1897. [41] New York Med. Rec., April 25, 1896. [42] Allbutt's System, vol. i. [43] Virch. Arch., Nov., 1896; Brit. Med. Jour., Dec. 19, 1897. [44] Lancet, Nov. 21, 1898.

